# An Essay on Diphtheria

**Published:** 1861-02

**Authors:** Henry W. Davis

**Affiliations:** Paris, Illinois


### AN ESSAY ON DIPHTHERIA.

BY HENRY W. DAVIS, M. D., OF PARIS, ILLINOIS.

Diphtheria or Diphtherite has occupied much space in the Medical Journals, and engaged much of the attention of the medical world during the past year. In regard to no affection has such a variety of opinions prevailed; and I am very certain that no one disease has had more advocates for varied and specified treatment. The Public Journals have as usual contributed their share of specifics; while our Medical Literature has been *sandwiched* by contributions from gentlemen who met the disease with the various surroundings, which not only control and modify its action, but exert a powerful influence in directing the treatment. Paris, Edgar Co., Illinois, was the first point west of Xenia, Ohio, at which the disease made its appearance; and as it was marked by a peculiar fatality at its first onset; and as it has been present during each succeeding year since 1853, sometimes in the form of an epidemic, I think an outline of the characters of the disease, embodying its pathology and treatment, may not be without interest or profit. We have read of epidemics of diphtheria in which the disease was controlled, and patients cured by the employment of a single medicinal agent; of others where cures were effected, and cases recovered under various constitutional remedies, without any, or but little attention having been paid to the effects of the disease as manifested in the throat. Such was not the kind of diphtheria brought under our observation, and such is certainly not the kind of treatment which a sad experience would induce us to recommend.

That diphtheria is a blood lesion, manifesting its presence not only in the throat, but upon all mucous surfaces, I am free to admit, but that it is the blood lesion which destroys the life of the patient, I am not willing to endorse. I conceive that a strong analogy exists in the pseudo membranous formation in dysentery, croup, bronchitis, and the same formation which appears in diphtheria, and in fact between the latter and all the plastic exudations peculiar to inflammatory affections of the mucous membranes.

Why it is that at one time we have an epidemic of dysentery, with a discharge of false membrane from the bowels, and at another a manifestation of apparently the same affection in the throat, I am unable to state. I leave the solution of that puzzle to those who are more familiar with the laws which control epidemics.

It is conceded, however, that when any general morbific cause prevails, nature will, after exerting the "vis medicatrix naturæ," succumb in that portion of the organism previously debilitated, or upon which the poison most readily exerts its influence; primary causes may exist which direct or locate the effects of the poison at different points; and thus it is in my opinion, why we have the same affection involving different parts, at different seasons, and under different miasmatic influences.

Whether the disease under consideration is purely sthenic or asthenic, I am not prepared to say, as it occurs in every variety of systemic condition, and appears to hold the laws of health in defiance. This doubt is predicated on the apparently opposite state of the system, in different subjects, at the time of an attack. Were I to judge from the effects of the remedies which have been most potent in my hands, and those of my brother practitioners in this locality, I should say that it is purely asthenic in its character. This opinion will, I think, be verified when I come to speak of the treatment, although that will not place it beyond a doubt. I shall speak first, in relation to the manner and place in which the disease manifests itself; or rather of that point of manifestation to which our attention is first directed. This is the upper portion of the throat. I cannot, in giving a history of diphtheria, go beyond that point when the discovery is made of false membrane upon the anterior surface of the tonsils, and free end of the uvula. The appearance of the pseudo membranous formation can be compared to nothing, more like, than the hard boiled white of an egg. The color may vary from a pearly white to a dull yellowish hue; a variation which may be accounted for by the fact, that the deviation from the white is always accompanied with a relative degree of hepatic derangement. There is no marked amount of œdema, or inflammation in the neighboring parts. The exudation looks as if it was stuck in to the mucous membrane, without any connection, pathologically or anatomically, with the parts beneath. Patients generally make little or no complaint of soreness of the throat, which may be attributed to the fact, that what ever of irritation may exist, is protected from additional irritation from the passage of food by the false membrane; so that in many cases, the disease may have approached the "Partæ Vatæ" long before its presence was suspected; and the first note of warning was the hoarse croupy cough, resulting from the presence or approach of the disease in, or towards the larynx. There may be some swelling of the sub-maxillary, sub-lingual and parotid glands, accompanying the disease, or preceding it; but not always is this the case. Whether or not, there are any appearances existing in the throat, prior to the formation of false membrane, which would forewarn us of an attack of diphtheria, I do not know, it comes unannounced; and when discovered, it demands all that skill and attention can bestow.

We have *read* of *one* who treated the disease solely with *Quinine*, losing only one in fifty. We admire the heroism indicated by the *dose* and the *faith*, and wonder at the success; but our patients die often before cinchonism would be produced by the largest dose of the salt. Another commends to us the use of salt and water as a gargle, and professes to have cured all his patients by its use alone. His patients were unlike ours, and the disease which succumbed to such a remedy was certainly different in character from that which we have had to encounter; as, in our hands, the most potent local applications failed in many cases, until a natural mode of using them was adopted. The above mentioned extremely simple methods of treating the disease, *may have been successful*; and were they not, still only negative in their character, little harm would be done, and no injury result from their promulgation and commendation. But they stop not on the side of negation; as, by misleading the inexperienced and unwary practitioner, they become positively hurtful, and unless fully sustained by facts, should receive as they deserve, the strongest reprehension. I would call in question no man's judgement or veracity, but he who writes a history of a disease which, under all circumstances, no matter how favorable, is marked by an unusual fatality, should be at least cautious in recommending the potency of any remedy which has nothing connected with it to sustain the laudations bestowed upon it. The disease is one which every practitioner in the west may be called upon to treat, and his first essay may be against an epidemic characterized by unwonted virulence. Its formidableness is testified to by the number of victims already placed to its credit; and it seems to me that the off hand suggestions, emanating from sources endorsed by unprecedented experience and *luck*, or sustained only by a *brace of fatal cases*, cannot be too much condemned, or received with too much caution. The disease may come upon the isolated practitioner, as it has come upon us, with all its terrible features in attendance, followed by dissolution so rapid, that the physician not only feel powerless to do good, but stands appalled at its ravages; under such circumstances, I do not believe that any one constitutional remedy is a specific; and feel fully warranted in saying that no reliance can be placed in any one systemic agent. I propose to speak of the affection as it appeared among us; the success which attended our first efforts to control it, and the means by which we eventually were enabled to cure many cases.

I know that in no locality, and under no circumstances, could it have existed in a more formidable form. For a long time diphtheria was a synonym for death; and it was not until 1855 that the rationale of the treatment was forced upon us by the powerlessness of all means hitherto used, and which compelled a resort to something more effectual, either in its nature or application.

The first cases which fell under my observation in 1854, attracted no attention, save that excited by their singularity; nothing of the kind having been seen previously by me. The small amount of constitutional disturbances; the slight local irritation or inflammation; the absence of complaint with regard to the throat, and the little apparent demand for the use of remedies, were all calculated to lead the casual observer astray, and force the young practitioner to acknowledge that both skill and remedies were powerless and inert. Yet, when one after another died, in spite of the best and most active treatment then employed, the disease rapidly became a source of more alarm than curiosity.

Death followed the invasion of diptherite in the throat with a celerity that seemed to defy the most skillful use of remedies; and although our most reliable tonics, alteratives and caustics were brought to bear, no good effect followed; yet I am satisfied that the failure originated not in the means, but in the applicant. Under these circumstances the disease became sufficiently alarming, but less so, from the fact, that it was considered non-contagious. In some few families there were several cases, but as a general rule, but one member was attacked, although surrounded by the same morbific causes. The victim was not the subject of any debilitating influences that were recognizable, but the disease appeared to display, in its selection, the most erratic disposition. So marked was this feature, that no one thought of making contagiousness one of its characteristics. It was then regarded as a purely local affection, demanding nothing beyond local remedies, so far as the treatment of the special local lesion was concerned.

In many, if not all the cases, malarious complications presented themselves, which succumbed to antiperiodic remedies, but the remedy did nothing towards curing the affection in the throat, thus proving that the disease was not only independent of the poison producing ague, but of the remedy itself, and during the first season of its manifestation, it only ceased to be fatal when it ceased to appear.

In tracing the history of the disease as it appeared among us, we find that during those seasons when we were wholly exempt from it, we were visited by epidemics affecting one or the other portions of the mucous tissues. During the prevalence of dysentery in 1855, there were no cases of diptherite. The earliest cases in 1854 were characterized by much sloughing after the false membrane gave way, as if the whole parts beneath were destroyed prior to the yielding of pseudo membranous formation. This peculiarity attending some cases gave rise to a differential diagnosis; and by many who saw them, it was regarded as ulcerated, or putred sore throat, and treated accordingly.

The throat presented a horrible appearance, and no remedy, as then applied, seemed to exercise any influence whatever Our patients passed rapidly from worse to worst, and the disease stalked beyond our skill, side by side with death. In other cases there was no sloughing at any stage of the disease, and the mucous surface presented little or no change after the disappearance of the diptherite. I believe that those cases where sloughing ensues, belong to the strictly asthenic class, and the others to the sthenic, if the word sthenic is at all compatible with diphtheria. Since 1855 we have had an annual visitation of diptherite in the throat, to a greater or less extent, present only during the absence of other *diptheritic* affections of the mucous membranes; and I must say that our success in its treatment has been much more decided. There is some difference in the nature of the local appliances, but they all tend to the same end, and are used for the accomplishment of the same object; and although the disease is a source of much dread, it is at least now within the range of rational and frequently successful treatment. In many, if not all the modes of managing a case of diphtheria, the medication has been predicated upon the fact conceded that it is a blood lesion, and remedies are directed to the removal of the poison from the system, while little or no attention is paid to the deposit in the throat. Our patients did not die of diphtheria, they were killed by diptherite. We do not think that in any case the blood lesion destroys life, but it is the result of that lesion manifesting itself in the throat, and producing occlusion of the air passages. Our cases therefore would not, and do not recover from any general or systemic treatments, from the fact that they die before the remedy could produce the desired effect. When the result of a disease destroys life in four, eight or twelve hours after discovery, it seems to me that any medicine relied upon to assist nature in eliminating the poison from the system, and thereby effect a cure, is certainly a *rope of sand.*

It was thus, and with such fatal and rapid effects, that the disease manifested itself with us; and it is therefore not strange, if after wading through page after page of essays on diphtheria, we should be astonished at finding no reccommendation of any agent, or agencies, to stay the spread of diptherite in the throat. All that could be gleaned was a recommendation to "buy the lock after the horse was gone." I only allude to the papers I have read, and they are not a few. It may be that the suggestions designed to be embodied in this paper, have already been better completed by him who saw the disease as we saw it, and who discovered earlier, that the way to cure *diphtheria* is by curing *diptherite.*

This apparent paradoxical assertion will, I think, admit of an orthodox explanation. Diptherite is first discovered, in a great majority of cases, upon the tonsils, and free end of the uvula. In rare cases, the buccal and labial membranes were found with patches on them; but its favorite habitat is upon the first named parts. Its tendency is downwards and backwards; and in this it differs from its kindred affection, pseudo membraneous croup, which starts from the air passages and travels upwards. If the discovery is made prior to the time of its extension to the posterior fauces, or on the epiglottis, it is amenable to treatment with some show of success. If the glottis, larynx, and trachea, are involved, it is at least a matter of exceeding doubt. It has been claimed that a successful termination has followed treatment, after the last mentioned parts were involved, but they are certainly exceptions to the rule; and I must confess that my faith is predicated not upon any success of my own, but upon that of my brother practitioners, whose skill and judgment are unquestioned.—Of the evidence of its presence in the air passages, and the treatment under the circumstances, I will speak fully before I have done. In speaking of the general treatment, we would endorse that which is of a tonic and alterative character, in so far as it has a tendency to support the vital powers, and assist in removing the blood lesion; but with the pathological manifestations which accompany the disease, we would deprecate the idea of relying on it alone, as it is less than a *slender reed.*

I said previously, and I again repeat it, that our patients die from the mechanical obstructions of the air passages, which produces suffocation; and that the cause, proximate or remote, did in no case destroy life. I do not think there is any organ or organs essential to life, which will yield to the influence of the poison of diphtheria, and produce dissolution; but there is one point which must be carefully guarded from its encroachments, and sufficiently protected against its attack, or your patients will, in a majority of cases, die. If this be true, the first duty forced upon the medical attendant, is the prevention of the extension of the disease downwards or backwards in the fauces, or from the anterior portion of the throat to the air passages. The invasion of the air passages is the *fons et origo* of that which induces death. This is the point which must be successfully protected, or your patients are always in danger, and the chances strongly against them. If you can use applications locally, which will prevent the formation of diphtheria between the tonsils and glottis, and thus prolong the life of your patient, I believe the disease will

*run its course*, and under the efforts of nature, the poison be eliminated from the system, and patients recover. Unless this is done, and that successfully, I believe that were all the systemic remedies in the Materia Medica brought to bear in a single case, not one would have a single chance for certain escape. If the ideas I have endeavored to convey, be correct, and the result of the disease as I have stated, true, certainly the suggestions relative to the treatment are based upon at least common sense; and I am sure it is the course *we* were compelled to pursue, after having been drawn from one resource to another by the fatality of the disease. Our belief in its asthenic character prompted an active tonic treatment, and although the degree of asthenia varied largely in different cases, yet we thought that in each one the prompt use of Quinine, wine and nourishing food, as supporters to, and Iodide and Chlorate of Potassa as alteraters of, the system, were indicated. I have been induced to admit the terms sthenic and asthenic, from respect to older practitioners, who classify it accordingly; yet I think when the wide distinction is drawn between the two phases of the disease, both of which demand, so far as our experience shows, a similar course of medication, it were well to drop them, and use the phrase *malignant* and *non-malignant*, as they certainly are the most expressive. The restoration and sustenance of the vital power was always a leading object, second only to arresting of the formation of false membrane in the posterior fauces and the larynx. For the accomplishment of this important end, I relied wholly upon the officinal Tincture of Iodine, applied with a probang. The object in view was the stimulation of the part, where the membrane was not already formed, and the application of a remedy which not only removed the semi-formed membrane, if it existed, but which, being absorbed, acted upon and altered the secretion from the vessels beneath. By this means we retain the diphtherite in "statu quo," and prevent its encroachments upon those parts of the organism where its approach is most to be dreaded, and where, once fully formed, it must in many cases prove fatal.

This is the BASIS of the only successful treatment of the disease as it prevails with us. It is therefore of the highest import that your remedy should be applied to the throat: and while the formation of false membrane is stayed in the anterior fauces, you may get time to operate on the blood lesion, if such a consummation is really essential to a cure. It has been suggested by one author, "that where early medication is resorted to, the disease may be apparently cured by absorbtion, but eventually re-appears and destroys life by operating upon a system already debilitated by the previous attack." I must confess that the idea of false membrane being absorbed, is with difficulty reconciled with the character of the formation, and I certainly have no testimony to offer to sustain the notion, or even give it the shade of plausibility. The idea of *real absorption* of *disease* is not only unmeaning, but ridiculous and preposterous. That the disease may be eliminated from the system, by the efforts of nature, is, I believe more than probable; and it may be that the effects would a second time appear, if the first manifestation of it was overcome by the recuperative power of the system, provided the poison was still in the blood. But I cannot see the force of the *idea* that a disease is *absorbed*, when it is admitted that its local habitation is the blood system. I believe that nature will do much, if not all, in curing the disease, if we can keep open the portals through which the supplies necessary to vitality are kept up; yet I would be unwilling to risk a case in the hands of nature alone, even after I had met the affection promptly in the throat. In no case that I have met, has the use of Quinine, Potassa, and the usual tonics and alteratives, been contraindicated; always, however, following an expectant course with regard to any symptoms that might arise indicating a special lesion, or constitutional derangement. It may be that in some cases the patient got well in spite of the constitutional tonic and alterative treatment; but I am satisfied that in no case would recovery have followed had not prompt and effective means been directed to the throat. I have thus endeavored to give an idea of the specific treatment which is our *sheet anchor* in controlling the disease. In commending this course I am sustained by Drs. Ten-Brook and York, who treated the disease in the same locality, and under the same circumstances. Dr. Ten Brook relies upon a strong solution of nitrate of silver, to arrest the spread of diptherite, applying it with a view to the accomplishment of the same end, which induces Dr. York and myself to use the Tr. of Iodine. I would give the iodine preference, because of its known action upon the absorbents, and would use it in diphtheria with the design of producing a like result, as when applying it to the cuticle, to arrest the spread of erysipelatous inflammation; and I must say that the nitrate of silver in my hands, has not proved as efficient, or as worthy of trust, as the high commendations of its endorsers would have led us to expect. The iodine is applied with a sponge saturated with the tincture, and to be of any benefit should be thrust firmly and far down in the posterior fauces. The limited strangulation of the patient will indicate the certainty of the remedy having reached the desired point.

If what I have written with regard to the disease is understood and appreciated, it will be manifest that an early discovery of the affection in the throat is essential to successful treatment. I would credit success in almost all cases to a diagnostic of diptherite in the anterior fauces. Some cases are reported of recovery when the larnyx and trachea were involved. This brings me to the consideration of one phase of the disease in connection with which I have no experience; still, as it may be an accompaniment of every case, I relate it here with the assurance that the authority comes from one, than whom no closer observer, or shrewder diagnostician is to be found in the state. Dr. York says "that when the false membrane has disappeared from view, and is no longer observable in the fauces, having been drawn before your remedies; and when aphonia is present accompanied by a stridulous cough, you may be sure that the larynx and trachea are the seat of fully formed, semi-organized diphtherite. Under these circumstances it is not judicious to use caustics or gargles in any shape, as they only add to the local irritation, and frequently hasten a fatal termination. There is no indication for the use of the probang when there is nothing visable to operate on. Under such circumstances, reliance is to be placed upon tonics, anodynes, stimulants and the free use of chlorate of potassa, internally and as a gargle, with the occasional administration of an alum emetic, if the strength of the patient will not allow the expulsion of the secretions in the trachea by voluntary effort. It is in these cases that you are compelled to rely upon the efforts of nature in combination with a systemic treatment; and local applications so far from doing good become really hurtful." In connection with the above statement, relative to the disease in the air passages, the evidences thereof, and the proposed treatment, I would suggest that in some cases where all the indications of a tracheal invasion were present, I have not looked upon them as sure prognostics, that such was the fact. In those cases of recovery where there was an entire disappearance of the formation from the eye, followed by aphonia, sibilant rale, I have not attributed the changes to the presence of diptherite, but to congestion, resulting from its near approach, or to the irritation produced by local remedies. It may be that I was wrong, and that the disease was really present; but I thought I marked a distinction between the vocal sounds, in those cases where I was satisfied it was in the air tubes, and which always resulted fatally, and in those where the disturbance resulted from thickening of the vocal chords from œdema. Where diptherite is deposited in the larynx, the vocal sound is always preceded and succeeded by a peculiar whistling sound which cannot be mistaken, and is as easily recognized as the cough in croup, while, where the traction is occasioned by irritation, the voice, though hoarse, is characterized by a uniform rhythm. I have not hesitated in these cases to still continue the use of the iodine, under the belief, always that the aphonia was the result of contiguous irritation, and from the presence of false membrane in the air passages. But admitting that the symptoms were pathogomonic of the presence of diptherite, may there not be an indication still for the use of the alterative application, with the same view that induced us to apply it to the throat above. The characteristics of the disease will aid us in giving plausibility to such a course. We are satisfied that, from the little complaint relative to the throat made by the patient, the false membrane protects it from irritation by the passage of food. May not the same cause, when present on, and in the glottis and epiglottis, operate so as to destroy the sensibility of the part, and admit a portion of the remedy into the larynx and trachea, and thereby produce the same beneficial effect exerted on the parts above? This has been the reason which justified me in the use of the iodine after the disappearance of the membrane from view, and the inauguration of those symptoms which would indicate its presence below, without, however, as stated previously, being convinced that the disease was really present. I would strongly insist, in cases where aphonia is present, upon the use of the tonic and alterative treatment. The use of the local applications must be left to the judgement of the physician, who will of course be guided by the circumstances attending the case. In all stages of the disease, and under all constitutional modifications thereof, the medical attendants cannot err in exhibiting each and every remedy that tends to sustain life, in the employment of hygienic precautions; but all are, I conceive, secondary to the attention which must be directed to the throat. In classifying the disease I spoke of the malignant and non-malignant forms. It was an experience with the first that prompted the writing of the paper. We met the latter as exceptional cases, and they yielded to local applications alone in the majority of instances. There is more constitutional disturbance attending the malignant form of the disease, and a greater aptitude towards swelling of cervical glands; but in many cases, there is no greater amount of such indications than attends the non-malignant. It would be hard indeed, to say to which form of the disease belongs the greater amount of systemic derangement in the early stages. So insidious is its approach, and so rapid the passage to the condition of complete adynamia or prostration, that but little time is afforded to note the symptoms which attend a case in its descending stages.

In a resume of the ideas embodied in this paper, as bearing upon the treatment, I would say that, close attention to the throat of all children who are within range of the disease—as *all* are liable to an attack ; the prompt application of iodine or nitrate of silver, between the part where the exudation is already formed, and the epiglottis ; the use of the application not oftener than once in twenty-four hours, are of the highest import. If aphonia with other indications exist, denoting the presence of the exudation in the air passages, the use of local remedies must be employed or discontinued, as the judgement of the medical attendant shall decide ; but experience commends a generous diet of essence of beef, eggs, milk, with the constant rational exhibition of that tonic which shall be best received by the stomach.

In submitting this paper, I have avoided, as far as possible, any indulgence in theoretical speculations. I have written at length, but have endeavored to impress forcibly the few important ideas which were wanting to fill the gap found in all the papers I have read. I know, if the germ can be culled, that the suggestions will be of no little importance to those who are likely to encounter the disease under the same influences, where the treatment recommended was, and is, successful. It is our best, and only reliance, and without it we would feel indeed powerless.

The topography of our Diphtheria may not be without interest. Paris is situated in the Eastern part of Illinois, within ten miles of the boundary line. It is on the edge of the grand prairie, and immediately adjoining the belt of timber which extends to the Wabash River. The country is pretty equally divided between prairie and timber. The first is more undulating than usual, from the fact that several small streams which empty into the Wabash, take their rise on it ; while the latter is considerably broken because of the streams running through it. About one-third of the country is under cultivation, while the rest is being rapidly enclosed. Its latitude is between 39° and 40° N., and Longitude 88° and 89° W. The disease was for a long time confined to the town, and since its appearance beyond its limits, the prairie seems to afford the elements for the development to a greater de gree than the timber. Like all diseases incident to this locality, it is subject to malarious complications, and it is rarely that we meet a case, but that we anticipate a duplication of it in the neighborhood.

